# The psychological profile of adolescents with anorexia and implications for treatment

**DOI:** 10.1186/2050-2974-2-S1-P9

**Published:** 2014-11-24

**Authors:** Stephanie R Damiano, Linsey Atkins, John Reece

**Affiliations:** 1 School of Psychological Science, La Trobe University, Melbourne, Australia; 2 Hope Family Clinic, Canberra, Australia; 3 RMIT University, Melbourne, Australia

## 

Anorexia Nervosa (AN) is associated with a range of physical and psychological symptoms that at times are perplexing for young people and their families to understand. For clinicians and patients, understanding the patient's psychological impairment can impact treatment engagement and outcome. The aim of this study was to explore the psychological profile of adolescent females with AN, including the relationships between eating symptomatology, general psychopathology, and social functioning. A clinical audit was conducted on the mental health files of 39 adolescent females with AN, aged between 13 and 18 years. A number of psychological measures were completed at their diagnostic assessment. Patients reported high levels of ineffectiveness, interpersonal problems, and general psychological maladjustment. Higher levels of eating symptomatology were related to higher levels of anxiety, depression, social stress, interpersonal problems, and lower self-esteem. Perceived ineffectiveness was strongly related to interpersonal problems and lower self-esteem. Patients who purged or reported suicidal ideation reported greater psychological maladjustment and interpersonal problems. These findings have implications for the potential inclusion of psychoeducation in the treatment of AN to help young people and their families understand how the illness impacts on psychological and social functioning.

